# Gestation-Based Viability–Difficult Decisions with Far-Reaching Consequences

**DOI:** 10.3390/children8070593

**Published:** 2021-07-13

**Authors:** Sumesh Thomas, Elizabeth Asztalos

**Affiliations:** 1 Department of Pediatrics, Section of Neonatology, University of Calgary, C536-1403 29St Nw, Calgary, AB T2N 2T9, Canada; 2 Department of Newborn and Developmental Paediatrics, Sunnybrook Health Sciences Centre, University of Toronto, M4-230, 2075 Bayview Ave., Toronto, ON M4N 3M5, Canada; elizabeth.asztalos@sunnybrook.ca

**Keywords:** viability, prematurity, gestational maturity, counseling

## Abstract

Most clinicians rely on outcome data based on completed weeks of gestational of fetal maturity for antenatal and postnatal counseling, especially for preterm infants born at the margins of viability. Contemporary estimation of gestational maturity, based on ultrasounds, relies on the use of first-trimester scans, which offer an accuracy of ±3–7 days, and depend on the timing of the scans and the measurements used in the calculations. Most published literature on the outcomes of babies born prematurely have reported on short- and long-term outcomes based on completed gestational weeks of fetal maturity at birth. These outcome data change significantly from one week to the next, especially around the margin of gestational viability. With a change in approach solely from decisions based on survival, to disability-free survival and long-term functional outcomes, the complexity of the parental and care provider’s decision-making in the perinatal and postnatal period for babies born at less than 25 weeks gestation remains challenging. While sustaining life following birth at the margins of viability remains our priority—identifying and mitigating risks associated with extremely preterm birth begins in the perinatal period. The challenge of supporting the normal maturation of these babies postnatally has far-reaching consequences and depends on our ability to sustain life while optimizing growth, nutrition, and the repair of organs compromised by the consequences of preterm birth. This article aims to explore the ethical and medical complexities of contemporary decision-making in the perinatal and postnatal periods. We identify gaps in our current knowledge of this topic and suggest areas for future research, while offering a perspective for future collaborative decision-making and care for babies born at the margins of viability.

## 1. Historical Perspective

Over the last 50 years, improvements in the survival and outcomes following preterm birth have changed significantly. While improvements in obstetric care have contributed to improvements in premature infant survival, the incidence of preterm birth continues to rise globally [1]. The incidence of live births at 22–25 weeks of gestation is reported at 4.7 per 1000 births in the USA (2014), 3.3 in Canada (2009–2014), 3.2 in the UK (2014–2015), 1.6 in Sweden (2011–2014), and 1.3 in Japan (2014–2015) [2].

**Figure 1:**
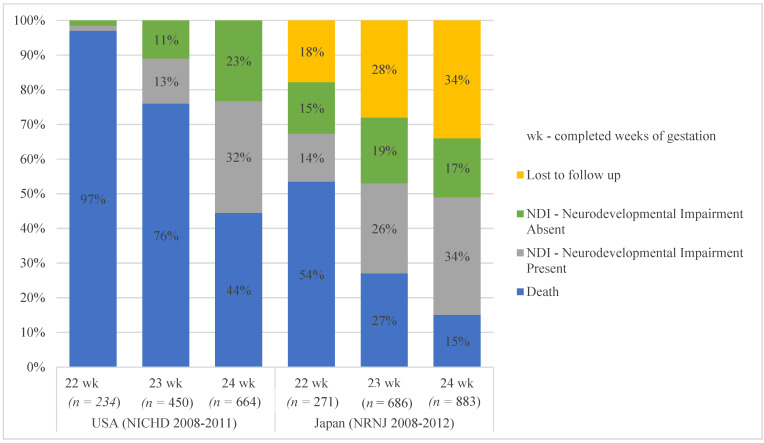
Outcome reports from multicenter databases.

In the 1960s, delivery before 28 weeks completed gestation was considered ‘previable’; however, by the 1990s, about 50% of babies born at 24 weeks survived with neonatal intensive care [3]. Over the last two decades, further improvements in survival and functional outcomes of babies born at 24 weeks gestation has led to parents and care providers to offer active interventions for babies born at 23 and 22 weeks of gestational maturity. Outcome reports from multicenter databases (i.e., from the USA, Eunice Kennedy Shriver National Institute of Child Health and Human Development (NICHD) 2008–2011; and the Neonatal Research Network of Japan (NRNJ) 2008–2012) show variations in outcome for babies born between 22 and 24 weeks gestation, with mortality rates of 50–90%, with birth at 22 weeks gestation, over a similar time frame (Figure 1) [4,5,6].

**Figure 2:**
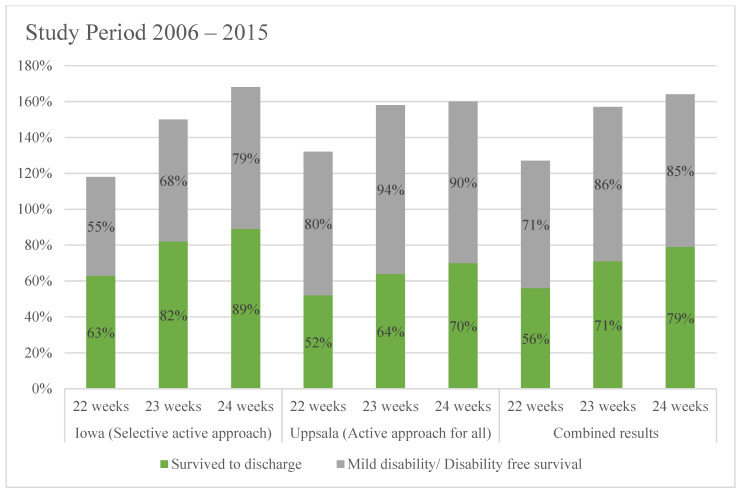
Comparative perspective for outcome at the margins of viability.

A recent publication from Uppsala, Sweden, reports 50% survival of babies born at 22 weeks gestation with over 50% of survivors reported as unimpaired at 30 months of age, with a uniform approach of offering active perinatal and neonatal interventions for babies born beyond 22 weeks gestation between 2006 and 2015 [7]. A similar publication (over the same period) from Iowa, USA, offering a selective approach based on parental wishes, reported 63% survival at 22 weeks gestation [8]. These two sites offer a comparative perspective for outcome at the margins of viability, although the number of infants treated was relatively small, the periods were similar, with potentially fewer variations in practice that influenced multisite databases with similar information (Figure 2).

The attitudes of care providers towards the outcomes of babies born at the margins of viability were shown to influence the perinatal care of pregnancies at 22–24 weeks gestation and subsequent neonatal outcomes [8,9,10,11,12,13,14,15,16,17]. These attitudes are however changing, as outcome data from recent publications suggest survival and outcome data for babies born at 22 weeks gestation—similar to what was achieved in the early 1990s with babies born at 24 weeks gestation [18]. This gradual shift of approach in care will likely continue with promising data showing early disability-free survival beyond 22 weeks gestation. Recognition of this change in practice has led to the publication of consensus statements, regarding the approach to pregnancies and births at the margins of viability [19].

## 2. Gestational Maturity

The short- and long-term outcomes for fetuses born at the margins of viability depend on multiple factors, including gestational maturity, fetal weight, fetal wellbeing, maternal health, fetal and maternal co-morbidities, available resources, social and economic considerations, societal expectations, ethical and moral considerations, values, and attitudes of care providers and families. Gestational maturity however remains the starting point for decisions related to viability for potential births between 22 and 24 weeks.

The Canadian Pediatric Society and Nuffield Council on Bioethics offer recommendations for the antenatal counseling and management of babies born before 26 weeks gestation. These recommendations are based on completed weeks of gestation of gestation maturity [20,21]. More commonly, gestational maturity is expressed in weeks and days for the calculation of postmenstrual age; for example, ‘24 weeks 5 days’. The majority of pregnancies rely on first-trimester ultrasound scans with an accuracy of ±3–7 days, to date gestational maturity. The reliability of these estimates also depend on the measurements used and the timing of the scans [22]. Therefore, except within the context of in-vitro fertilization (IVF), gestational maturity remains an estimate with a half- to a full-week of greater or lesser maturity at the time of discussions related to perinatal and neonatal care. Recognition of this uncertainty forms part of the complexity enshrouding discussions of prognosis related to the care of infants born at the margins of viability.

What is evident from large multicenter databases on neonatal outcomes is that change outcome changes significantly with each week of increasing gestational maturity, especially with preterm births before 26 weeks gestation. The Canadian Neonatal Network (CNN) reports survival at 31% below 23 completed weeks, 55% at 23 weeks, 72% at 24 weeks, 81% at 25 weeks, and 87% at 26 completed weeks [23]. With increasing gestational maturity, the incidence of three major neonatal morbidities ((1) severe neurological injury; (2) bronchopulmonary dysplasia (BPD); (3) and stage 3 or higher retinopathy of prematurity (ROP)) decreases [24]. These changes in incidence, from one 7-day period to the next, are quite dramatic, calling one to rethink the appropriateness of counseling based on completed gestational weeks of maturity.

Studies looking at neonatal outcomes with maturity changes of less than 7 days in babies born before 26 weeks gestation have shown that morbidity and mortality differ between the early and late parts of gestational week [25,26,27]. This calls into question the rational for making significant clinical decisions that have a significant impact on patients and their families, based on a seven-day gestational maturity basis. Fetal maturity and development is subject to multiple factors not limited to genetics, maternal health, and nutritional status, placental function, and environmental factors. The use of a seven-day or even a 24-h cycle to confidently estimate functional and structural maturity is questionable, as the process of organogenesis may not necessarily follow conventional systems of timing.

## 3. Counseling

Parents have expressed a need for clearer and consistent counseling with interpretable information to base life-altering decisions [28]. The use of completed weeks of gestational maturity at the margins of viability might simplify counseling for clinicians; however, the uncertainty associated with this approach will likely leave families with more questions than answers. There is a need for clearer and interpretable data available to healthcare providers and families, which take into account variables that influence the outcome, such as gestational maturity in days rather than weeks, the presence or absence of congenital anomalies, gender, estimated birthweight, singleton versus multiple pregnancies, surrogate markers of fetal wellbeing, presence or absence of chorioamnionitis, use of antenatal steroids, mode of delivery, and delivery at a perinatal facility.

Inaccuracy is the estimation of fetal weight at the margin viability within the delivery room, setting in, up to 62% of cases, was reported to influence perinatal care. Overestimation of fetal weight resulted in greater survival with active perinatal and neonatal interventions. Non-cephalic fetal presentations and decreased liquor volume tending to underestimate—while a high maternal body mass index tended to overestimate—fetal weight [29]. While survival of extremely premature babies weighing <500 g is reported, the incidence of major morbidity remains high in this group, irrespective of gestational maturity [30].

Most centers offering a selective approach with care provision at the margin of viability or within the so-called “gray zone” support collaborative decision-making with parents in the face of prognostic uncertainty [31]. An accurate estimate of gestational maturity and fetal wellbeing guides collaborative decision-making between parents and care providers. These discussions determine the expectations of families in making decisions on perinatal interventions, such as the initiation of interventions aimed at improving survival and neuroprotection for the newborn, the use of tocolytics, antenatal steroids, antibiotics, magnesium sulfate, mode of delivery, and the potential transfer of mothers to high-risk perinatal facilities for further counseling and potential delivery.

Antenatal counseling should also include potential challenges at resuscitation, such as the need for cardiac compression and epinephrine during resuscitation, which is known to worsen prognosis [32]. Discussions about the importance of neonatal nutrition, including the reliance on central lines for parenteral nutrition and preference for the use of human breast milk to feed the extremely preterm infant, emphasizes the importance of growth and nutrition in improving survival and functional outcomes. Despite successful resuscitation and admission to the neonatal intensive care unit, prediction of long-term outcomes during the neonatal period remains a challenge. The presence of significant intracranial hemorrhage, severe lung injury, renal impairment, and inflammatory conditions resulting in impaired physiology, requiring escalating levels of cardiorespiratory support, raise the likelihood of death and long-term disability. The absence of significant abnormalities on neuroimaging in the neonatal period, while reassuring, does not provide certainty of the absence of long-term neurodevelopmental disability [33].

Inevitable long-term follow-up is required to fully evaluate the impact of preterm birth in survivors. This uncertainty exists at the start of admission for neonatal intensive care and is likely to remain for several years after discharge from the hospital. Irrespective of short- or long-term outcomes, consistent, honest, and timely communication with families provide the basis for a truly collaborative partnership for decision-making and in optimizing care for vulnerable babies. Timely and compassionate communication offers the opportunity to review progress and reconsider treatment goals, especially in the face of significant deterioration with escalating levels of care, without clear evidence of benefit [34]. Such communication also mitigates parental dissatisfaction and potential conflict between parents and care providers [35]. For babies born at less than 24 weeks gestation, long-term outcomes are still being evaluated; discloser of this limitation should form part of antenatal and postnatal counseling.

## 4. Postnatal Care

While sustaining life following birth at the margins of viability remains a priority—identifying and mitigating risks associated with extremely preterm birth begins in the perinatal period. The postnatal care needs of extremely premature infants are unique, with the task of sustaining physiological stability within the context of extremely fragile and immature organ systems not readily functional for extrauterine life. Delivery at tertiary care perinatal facilities ensures ready access to infrastructure and resources required for caring for extremely premature low birthweight infants and mitigates additional risk associated with postnatal transportation of fragile babies. Care provided at birth centers where senior neonatal clinicians attend the delivery of babies born at the margins of viability, has reported better outcomes; however, this in itself was not proven to be a significant factor in determining the outcome [11]. Nonetheless, the attendance of a senior neonatal clinician was recommended by the British Association of Perinatal Medicine (BAPM) [36].

The need for transportation in the immediate postnatal period was shown to carry a worse prognosis, especially for premature babies. A large Canadian study of over 2000 babies born at less than 29 weeks showed poorer neurodevelopmental outcomes at 2 years of age in transported babies [37]. A similar study from the UK, comparing outcomes for premature inborn and outborn babies, also showed a similar risk, despite the administration of antenatal steroids [38]. The risk of intracranial bleeding is highest in the first 72 h, and with this, a greater risk of neurodevelopmental disability. The timing of most neonatal transports coincides with the period of greatest vulnerability for intraventricular bleeding. Birth at a facility without specialist equipment and teams skilled in the resuscitation and stabilization of extremely premature infants, condition at birth, increased handling, vibration, noise, and duration of transport, have all been implicated in the higher incidence of intraventricular bleeding [39,40]. Ideally, timely counseling of families in consultation with a neonatologist would help families with decisions regarding desired perinatal and neonatal interventions. With infants likely to be born at the margins of viability, administration of antenatal steroids, and in-utero transfer to tertiary care perinatal facility should be the priority when active care is desired.

Challenges in caring for extremely premature babies require extreme care in minimizing iatrogenic injury from birth. Effective implementation of lung and neuroprotective strategies from birth requires the presence of personnel experienced in the resuscitation and stabilization of extremely low birthweight and extremely premature babies. The challenge of supporting the normal maturation of these babies after birth has far-reaching consequences, and is dependent on our ability to sustain life while optimizing growth, nutrition, and repair of organs compromised by the consequences of preterm birth [41]. Promoting the use of the fortified donor and mother’s breast milk was shown to decrease the risk of necrotizing enterocolitis (NEC) without adversely impacting growth [42]. Vulnerability to significant neurological injury in the neonatal period along with life-limiting complications resulting in multi-organ dysfunction accounts for the most neonatal deaths. Studies have shown that the incidence of major neonatal morbidities (neurological injury, BPD, ROP, and necrotizing enterocolitis) increases with decreasing gestational maturity. Severe forms of these neonatal morbidities are known to contribute to the burden of long-term adverse outcomes [43]. Contemporary neonatal care inevitably treads a fine line between managing iatrogenic injury caused by the application of life-sustaining therapies and the preservation of life and long-term function.

Centers with long-standing expertise in the care of extremely premature infants have described unique approaches to the care of babies born at the extremes of viability. In Japan, the limits of gestational viability changed from 24 to 22 completed weeks in 1991. A publication in 2019 described a comprehensive approach to the care of extremely premature infants with emphasis on cardiovascular care, respiratory management, neuroprotective measures, nutritional support, and management of the risk of infections [44]; (Table 1). With this approach, the Neonatal Research Network of Japan reports on lower rates of mortality and severe intraventricular bleeding in comparison to similar databases from Canada, Sweden, Israel, the UK, Switzerland, Spain, and Australia [45]. They however reported a significantly higher incidence of retinopathy of prematurity requiring treatment.

## 5. Survival to Discharge

Comprehensive discharge planning and outpatient medical review are required for most extremely preterm infants on discharge from the neonatal intensive care environment. There is a significantly higher risk of readmission to the hospital following discharge in the first couple of years, especially for babies being discharged on supplemental home oxygen [46]. The risk of dying from the consequences of extreme prematurity is also greatest in these first two years of life [47].

At this time, there is limited information on the health outcomes of extremely premature babies born in the 1980s and 1990s who are beyond their third decade of life. This information is relevant to understanding the additional effect of aging on the health of patients who survived complications of prematurity at the start of life. Ideally, lifelong medical follow-up would be required to better evaluate age-related changes in physiological capacity and its influence on care needs and quality of life.

## 6. Ethics

Nonintervention at 22–24 weeks gestation would result in 100% mortality without the initiation of life-sustaining treatment, while recipients of active care inevitably require prolonged periods of intensive care support, with the potential for survivors to suffer short- and long-term morbidity. Arguments based on principles of ‘sanctity of life’ and ‘quality of life’ are intricately linked and yet have provided diametrically opposing arguments to decision making in medical care, since the outset of modern medicine, and the ability to preserve life with medical interventions.

Except with IVF pregnancies, gestational maturity assessed between 22 and 24 weeks gestation remains challenging, with an inaccuracy of a factor of ±3–7 days, depending on the methods used. It, therefore, stands to reason that a mother presenting at 23 completed weeks of gestation could indeed have a fetus that is at 22 weeks or 24 weeks gestation. It also stands to reason that the outcomes for babies born at the margins of viability are unlikely to be significantly different at the stroke of midnight from one gestational week to the next. Counseling and decisions taken based on this concept could defer depending on the interpretation of this reality. Furthermore, studies that have shown similar short-term outcomes at the early part of the gestational week compared to the latter part of the preceding week add to this conundrum. It is therefore imperative that parents are appropriately counseled regarding imprecisions with the assessment of gestation based on first-trimester ultrasonography.

Data from centers that practice a selective approach, based on parental wishes as well as from centers that have adopted an active approach for all, have shown that survival is indeed possible at gestation as early as 22–23 weeks, while follow-up data mostly report on neonatal morbidity and early childhood outcomes for this subset of babies. It is therefore difficult to propose a contrary quality of life argument for the non-initiation of active care at the current margins of viability if parents request active interventions. Having said that, during neonatal intensive care, decisions relating to goals of care may need to be revisited with parents, based on the severity of complications of prematurity when survival without severe disability is unlikely or medical interventions appear futile and death is inevitable.

Ethical principles of care at the extremes of viability rely largely on the interpretation of published literature. It is therefore important that centers that offer a selective or active approach in caring for babies born at these gestations offer comprehensive insights into the short- and long-term outcomes of these extremely vulnerable babies. This information will become increasingly relevant as the margins of viability creep toward lower gestational ages, as seen in the late 1980s and early 1990s. Individualized antenatal and postnatal risk profiles based on modifiable and non-modifiable factors would ideally guide the implementation of perinatal and postnatal care processes.

With most expectant parents instinctively more receptive to positive information, interpretation of unfavorable outcome information is subject to interpretational bias by parents, even in the face of irrefutable guarded prognosis [48]. The perception that care providers are overly pessimistic could lead to mistrust and challenges in finding common ground for constructive engagement of families as competent partners for collaborative decision-making with care providers. Nonetheless, a compassionate approach aimed at providing holistic care for the patient and family, based on clear and timely communication, fosters the principles of family-centered care and collaborative decision-making, especially in the face of medical uncertainty. This approach is likely to minimize conflict and improve satisfaction for parents and caregivers, irrespective of the short- or long-term outcome for the patient.

The interpretation of parental requests to ‘do everything’ to ensure the survival of their baby must be interpreted by clinicians within the context of acceptable evidence-based practices. Care must be taken to avoid the initiation of potentially harmful and or futile interventions that are likely to prolong biological life, while causing suffering without real benefit to the patient. Most medical licensing agencies empower healthcare providers to be under no obligation to offer futile interventions, and that doing so in the full knowledge of its futility could be contentious. The ‘escalator of neonatal intensive care’ has been used to describe progressive increments in interventions aimed at maintaining physiological parameters, with the inevitable progression towards demise. Collaborative decision-making among healthcare providers not only minimizes significant variations in care, it also ensures consistent information for families and potential safeguards against embarking on medically futile interventions.

## 7. Conclusions

With advances in neonatal care over the last 50 years, the survival of babies born at the margins of viability has become a reality in contemporary neonatology, with increasingly more centers reporting on the short-term outcomes of babies born between 22 and 24 weeks gestation. As healthcare providers, the onus is on us to provide patients and surrogate decision-makers information that is understandable and complete, highlighting areas of medical uncertainty. This is important in forming competent collaborative partnerships with families in care and decision-making. Antenatal and postnatal counseling at the margins of viability should take into account current limitations with the assessment of gestational maturity based on first trimester ultrasounds. This limitation should be disclosed at counseling.

As the viability of lower-order gestations becomes a moving target in contemporary neonatal care, there is a need for better assessment of fetal maturity and predictors of short- and long-term outcomes to guide future practices. Longer-term studies are still needed to fully understand the lifetime implications of extreme prematurity in survivors. As margins of viability continue to erode with the survival of babies born as early as 22 weeks gestation, and in the face of promising animal studies using artificial placental support [49], is it time to rethink extra uterine care for babies born before 24 weeks gestation?

## Figures and Tables

**Table 1:** Clinical approach to the management of extremely preterm infants in Japan.

Viability	Active Resuscitation of Infants at 22–23 Weeks Gestational Age
Circulatory Care	Guided by neonatologist-performed echocardiography
Respiratory care	Reliance on early invasive ventilation
Neuroprotection	(I) Minimal handling; (II) sedation for ventilated infants; (III) serial cranial ultrasounds
Nutritional care	(I) Promotion of breastfeeding; (II) early minimal enteral feeding; (III) routine use of glycerin enema; (IV) use of probiotics
Infection	(I) Gloves, masks, gowns for patient care; (II) serial CRP monitoring
